# A Fermentation State Marker Rule Design Task in Metabolic Engineering

**DOI:** 10.3390/bioengineering10121427

**Published:** 2023-12-15

**Authors:** Egils Stalidzans, Reinis Muiznieks, Konstantins Dubencovs, Elina Sile, Kristaps Berzins, Arturs Suleiko, Juris Vanags

**Affiliations:** 1 Institute of Microbiology and Biotechnology, University of Latvia, Jelgavas Street 1, LV-1004 Riga, Latvia; reinis.muiznieks@lu.lv (R.M.); kristaps.berzins@lu.lv (K.B.); 2 Bioreactors.net AS, Dzerbenes Street 27, LV-1006 Riga, Latviaelina.sile@rtu.lv (E.S.); arturs.suleiko@bioreactors.net (A.S.); juris.vanags@bioreactors.net (J.V.); 3 Laboratory of Bioengineering, Latvian State Institute of Wood Chemistry, Dzerbenes Street 27, LV-1006 Riga, Latvia

**Keywords:** marker rule, genome scale metabolic model, metabolic engineering, growth coupling, production envelope

## Abstract

There are several ways in which mathematical modeling is used in fermentation control, but mechanistic mathematical genome-scale models of metabolism within the cell have not been applied or implemented so far. As part of the metabolic engineering task setting, we propose that metabolite fluxes and/or biomass growth rate be used to search for a fermentation steady state marker rule. During fermentation, the bioreactor control system can automatically detect the desired steady state using a logical marker rule. The marker rule identification can be also integrated with the production growth coupling approach, as presented in this study. A design of strain with marker rule is demonstrated on genome scale metabolic model *iML1515* of *Escherichia coli* MG1655 proposing two gene deletions enabling a measurable marker rule for succinate production using glucose as a substrate. The marker rule example at glucose consumption 10.0 is: IF (specific growth rate μ is above 0.060 h^−1^, AND CO_2_ production under 1.0, AND ethanol production above 5.5), THEN succinate production is within the range 8.2–10, where all metabolic fluxes units are mmol ∗ gDW^−1^ ∗ h^−1^. An objective function for application in metabolic engineering, including productivity features and rule detecting sensor set characterizing parameters, is proposed. Two-phase approach to implementing marker rules in the cultivation control system is presented to avoid the need for a modeler during production.

## 1. Mathematic Models in Fermentation Process Control

There is a long history of mathematical modeling applied to fermentation control. The rapid development of batch technologies and automation methods facilitated mathematical modeling application for cultivation processes description and feed rate calculation.

When fed-batch mode is used to control a parameter—biomass growth rate, product biosynthesis, or substrate concentration—various mathematical models are used to calculate the profile. Mathematical models are applied to describe the relationships (correlations) between changes in the concentration of biomass, metabolites, and substrates in the cultivation medium. Fermentation process models can be divided into two main groups: white box models (WBM)—mechanistic models that use mass balance information in a single stoichiometric equation, and black box models (BBM)—empirical models that describe input–output relations without mechanistic interpretation [1]. Black box modeling is used in the development of soft sensors for process recognition [2,3].

Mechanistic models contain equations that describe changes in the concentration of biomass, substrates and volume of the medium, and also describe the dependencies of growth rates on the concentration of substrates or inhibitory factors [4]. One of the most popular and frequently used models is the Monod model [5]. The structured models take into account the internal structure of cells as well as the variation in their composition and morphology. These models enable prediction of the influence of various factors on the entire process [6].

The constraint-based genome scale stoichiometric modeling of metabolism [7,8] has been developing fast in the last decades, yet no application of genome scale stoichiometric models in the control of fermentation process is reported. The potential of increased mechanistic understanding of fermentation using cellular metabolism models [9] is determined by their mechanistic nature, relatively easy measurements of metabolites and applicability of mass conservation law [10]. In this study, we present and discuss novel application of mechanistic genome scale stoichiometric models of cellular metabolism to improve the fermentation control systems introducing steady state marker rules.

## 2. Applications of Mathematical Modeling in Metabolic Engineering

### 2.1. Mathematical Modeling of Metabolism

The implementation of mathematical modeling of metabolism has become increasingly popular in biotechnological applications moving from theoretical aspects towards implementation [11,12].

Mathematical modeling of cellular metabolism is applied in biotechnology mostly as (1) genome-scale constraint-based stoichiometric modeling of cellular metabolism steady state and (2) pathway scale ordinary differential equation (ODE)-based kinetic models [9]. The software tools for the two mentioned types of models are well developed and available to users. The constraint-based genome scale stoichiometric models of metabolism [7,8] can be simulated and optimized by COBRA toolbox v.3.0 with many supporting packages [13] by specialists with programming skills, while CNApy software (https://github.com/cnapy-org/CNApy, accessed on 29 November 2023) [14] can be applied also using the graphical user interface even by unexperienced users. In the field of ODE-based pathway scale kinetic models there are different Matlab and Python based tools for specialists with programing skills. COPASI (https://copasi.org/, accessed on 29 November 2023) [15,16] stands out as a two decades developed tool with graphical user interface and some supporting tools using COPASI functionality [17,18].

### 2.2. Metabolic Engineering Approach

The design of biotechnologically efficient strains by metabolic engineering [19,20] applying mathematical modeling and optimization approaches has proven to be practical. The trend of implementation of cellular engineering ideas and methods into education [21] makes the practical implementation of engineered strains more interpretable and applicable in the industry.

Metabolic engineering usually is applied to improve the productivity or yield of biotechnological production by deleting native reactions, inserting enzyme-coding genes from other organisms and/or over/underexpressing enzymes identified by optimization efforts. There are some metabolic engineering approaches that are applicable in specific task settings.

A growth coupling concept is used in metabolic engineering to determine what genetic manipulations will enable growth-coupled product synthesis the way that product becomes a mandatory by-product of growth [22].

Another specific application of metabolic engineering is sustainable metabolic engineering [23], where the sustainability factors of all incoming and outgoing fluxes are taken into account during design optimization [24].

These specific applications of metabolic engineering encourage further exploration of metabolic engineering application extensions.

## 3. Side-Task of Metabolic Engineering: Desired Steady State Marker Engineering

### 3.1. Measurable Steady State Marker Rules as Side-Task of Metabolic Engineering

In fermentation processes where the synthesis of the target product cannot be directly measured and the process can drift from the desired fermentation steady state, it would be advantageous to have some set measured values that can be used by the bioreactor control system to identify the desired steady state or deviation from it, which is very important for unstable fermentation processes.

We propose the inclusion of a desired steady state identifying marker rule search in the task setting of metabolic engineering. In parallel with improving fermentation productivity and/or yield, the marker rule can be identified. Marker rule identification can be combined with the production growth coupling approach, as presented in this study.

Logical marker rule can be interpreted also as a kind of soft sensor [25,26] where the meaning of the marker rule is derived from genome scale metabolic model optimization and has a mechanistic interpretation in contrast to soft sensors. There is also some similarity to biomarkers in medicine that could be sensed to identify cells’ state [27]. The diagnosis of a disease is positive if certain molecules are present or absent, or if they increase or decrease (as an analog of marker rule). These approaches, in contrast to the proposed marker rule design, do not use metabolic engineering for strain design.

The described growth-coupling approach (Section 2.2) can be applied to use the specific growth rate μ as desired metabolic steady state marker. A typical way to represent the feasible solution space in the growth coupling approach is to plot the growth rate vs. production rate. A wild-type strain solution space envelope usually has no product synthesis at maximal growth rate. Looking for gene deletions with a growth-coupling approach the aim is to have at least some product synthesis at the maximal growth rate expecting that the phenotype with the highest growth rate will dominate in the bioreactor due to faster growth and will inevitably produce the target metabolite [28]. It is also worthwhile to keep in mind that there are cases where growth cannot be coupled with production or the coupling is not attractive (weak coupling, low production rate a.s.o.) [22].

### 3.2. Example of Steady State Marker Rule

For illustration purposes we engineered genome scale metabolic model *iML1515* of *Escherichia coli* MG1655 to develop a measurable marker rule for succinate production using glucose as substrate with consumption rate 10.0 (mmol ∗ gDW^−1^ ∗ h^−1^).

#### 3.2.1. Specific Growth Rate as a Marker

For fermentation state marker rule illustration purposes in our study, we used an analog to the design #9031 proposed in the study of Muiznieks et al., [24] with two gene deletions: pgi (b4025) and atpG (b3733) in *E. coli* model *iML1515* [29]. We applied stoichiometric constraint-based genome-scale modeling [30] using the Matlab toolbox COBRA v3.0 [13] and growth-coupling packages optGene [31] and OptEnvelope [32]. Glucose consumption rate of 10.0 (mmol ∗ gDW^−1^ ∗ h^−1^) under anaerobic conditions is assumed during calculations. Corrections of flux values used in marker rules are required if glucose consumption differs.

**Figure 1:**
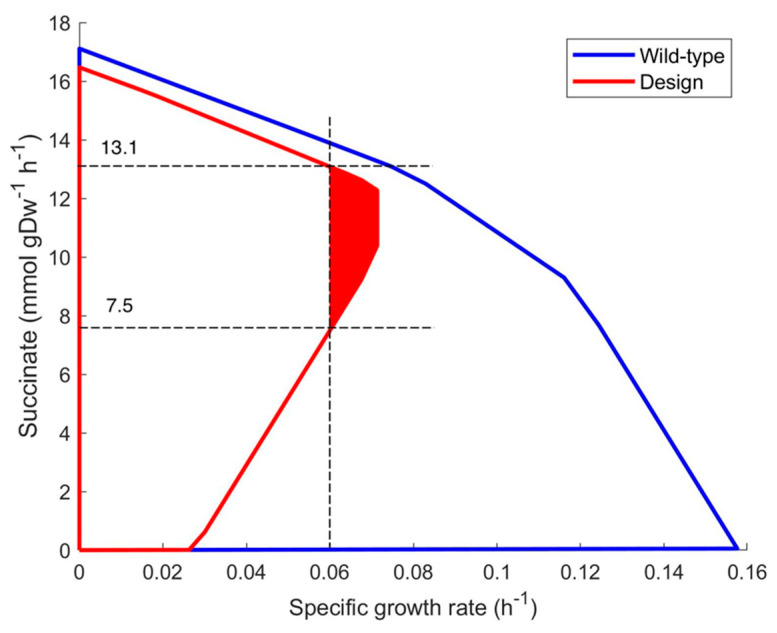
Growth coupling envelopes of wild-type strain *iML1515* model (blue line) and the same model with designed deletions of genes pgi (b4025) and atpG (b3733) (red line). The feasible steady state area indicates that succinate can be produced within the range 7.5–13.1 (mmol ∗ gDW^−1^ ∗ h^−1^) if μ is above 0.060 h^−1^ (colored in red).

In the growth coupled succinate production plot (Figure 1), the succinate productivity in the area of the maximal growth rate of wild-type strain has been zero while it is 10.3–12.3 (mmol ∗ gDW^−1^ ∗ h^−1^) for the designed strain due to the two suggested gene deletions.

As a consequence, the measurement of biomass growth rate can be used as a simple marker to determine succinate synthesis variability. In Figure 1, a growth rate above 0.06 h^−1^ implies that succinate synthesis should be within the range 7.5–13.1 (mmol ∗ gDW^−1^ ∗ h^−1^). Even if a higher growth rate (0.065 h^−1^) is observed, product synthesis should be within the range of 8.7–12.7 (mmol ∗ gDW^−1^ ∗ h^−1^). These numbers are valid, assuming that the genome-scale metabolic model is correct and that glucose consumption is 10.0 (mmol ∗ gDW^−1^ ∗ h^−1^) under anaerobic conditions.

The above-mentioned relation between biomass growth and product synthesis we propose to call a marker rule: conditions that determine the expected range of product synthesis flux depending on an online measurable value—a specific growth rate μ in this case.

The earlier mentioned relations in the production envelope can be expressed in a marker rule with metabolite flux values:

**Marker rule R1**
IF specific growth rate μ is above 0.060 h^−1^,
THEN succinate production is within the range of 7.5–13.1 (mmol ∗ gDW^−1^ ∗ h^−1^).
**Marker rule R2**
IF specific growth rate μ is above 0.065 h^−1^,
THEN succinate production is within the range of 8.7–12.7 (mmol ∗ gDW^−1^ ∗ h^−1^).

#### 3.2.2. Combination of Several Parameters into Marker Rule

As a marker, specific growth rate is applicable when product synthesis coupling with biomass is possible and the minimal productivity value is satisfactory. Unfortunately, that is not always the case. More complicated marker rules may help to deal with such cases or improve the biomass growth rate predictive value.

Another reason to make the rule more complicated is to exploit markers for higher production rate within the productivity range determined by biomass growth rate alone. Another advantage of additional parameters in the marker rule is increased reliability and stability of the rule. This is because fluctuations of one parameter can be compensated by the involvement of other parameters.

**Figure 2:**
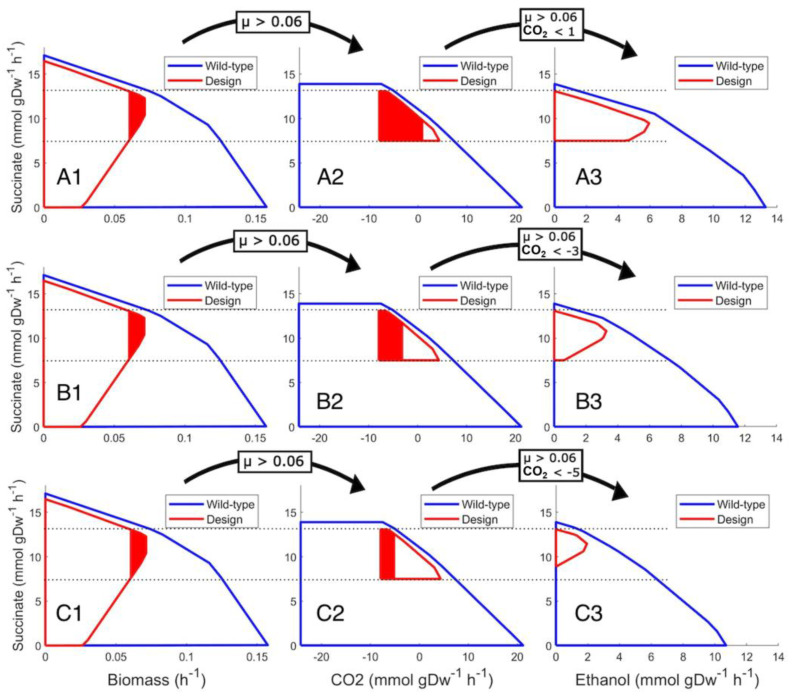
The effect of additional exchange metabolite flux inclusion on the marker rule. Wild type (blue line) and designed solution space envelopes (red line) illustrate three different marker rules (row A for rule R3, row B for rule R4 and row C for rule R5) at specific growth rate μ above 0.060 h^−1^ (**A1**–**C1**), depending on the exchange rate of CO_2_ (**A2**–**C2**) and ethanol production rate (**A3**–**C3**). The arrows above the figures indicate marker rule elements applied to the next plot. The red colored areas indicate the effect of marker rules applied for the next section.

The effect of more exchange fluxes implemented in marker rules can be visualized (Figure 2) and expressed as marker rules (rules C, D and E). As long as the specific growth rate is kept as in rule A and CO_2_ exchange and ethanol production are measured, the succinate production range can be narrowed, and the minimum production rate can be increased. In all sections of Figure 2, succinate production is on the “y” axis, while in sections A1–C1, the “x” axis is specific growth rate; in sections A2–C2, the “x” axis is CO_2_ exchange, and in sections A3–C3, there is ethanol production. That causes different envelope shapes and illustrates that different metabolic fluxes can be linked with production and used in marker rules.

In accordance with Figure 2, a set of marker rules with CO_2_ exchange and ethanol production, in addition to biomass, enable enhanced marker rules with increased succinate minimal production values:

**Marker rule R3** (row A in Figure 2)
IF specific growth rate μ is above 0.060 h^−1^,
AND CO_2_ production is under 1.0 (mmol ∗ gDW^−1^ ∗ h^−1^),
AND ethanol production is above 5.5 (mmol ∗ gDW^−1^ ∗ h^−1^),
THEN succinate production is within the range of 8.2–10 (mmol ∗ gDW^−1^ ∗ h^−1^).
**Marker rule R4** (row B in Figure 2)
IF specific growth rate μ is above 0.060 h^−1^,
AND CO_2_ consumption is at least 3.0 (mmol ∗ gDW^−1^ ∗ h^−1^),
AND ethanol production is above 3.0 (mmol ∗ gDW^−1^ ∗ h^−1^),
THEN succinate production is within the range of 10.0–11.2 (mmol ∗ gDW^−1^ ∗ h^−1^).
**Marker rule R5** (row C in Figure 2)
IF specific growth rate μ is above 0.060 h^−1^,
AND CO_2_ consumption is at least 5.0 (mmol ∗ gDW^−1^ ∗ h^−1^),
AND ethanol production is above 1.5 (mmol ∗ gDW^−1^ ∗ h^−1^),
THEN succinate production is within the range of 10.8–12.3 (mmol ∗ gDW^−1^ ∗ h^−1^).

**Figure 3:**
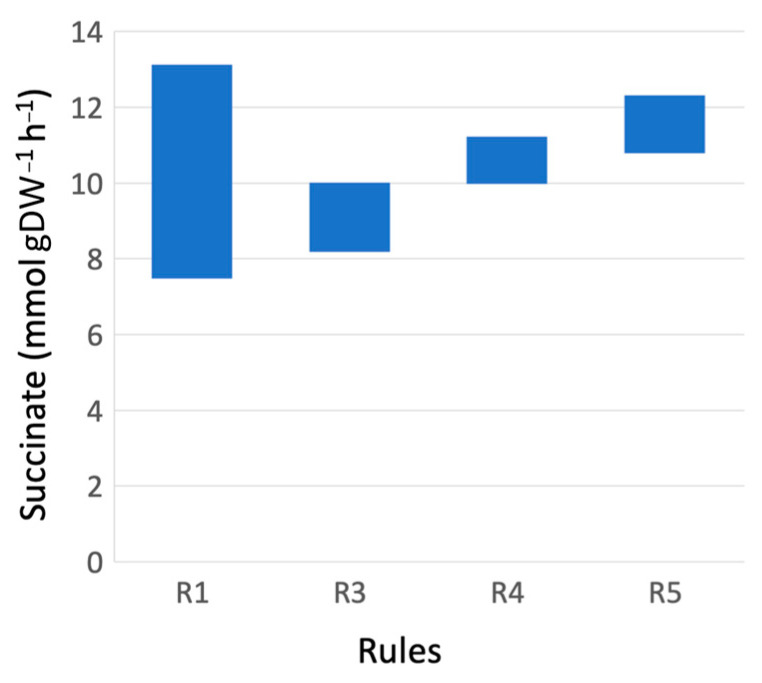
Succinate production range in case of rules R1, R3, R4 and R5 when the specific growth rate μ is above 0.060 h^−1^.

As a result, marker rules have improved the range of succinate production (Figure 3). A widely possible succinate production range, when just the specific growth rate is above 0.06 h^−1^ (rule R1), is narrowed by rules R3–R5 when increasing minimal succinate productivity.

In other strain designs, the rules and envelopes can be very different. Section 4 describes the concept of rule creation and ranking of designs automatically. Growth-coupled production has been used here in all marker rule examples, but metabolic state marker rules may even not include biomass growth rate due to the complexity of its measurement and correct interpretation.

## 4. Development of Marker Rules

The design of organism modifications with a convenient marker rule, which can be reliably observed at minimal cost, is a complicated task with many variables:

- Target product;
- Chassis organism;
- Reaction deletions, inserts or over/underexpression of native enzymes to determine marker rules;
- Sensors and measurement systems to check the marker rule;
- Media composition.

### 4.1. Determination of Measurable Parameters

It would be ideal if the marker rule parameters could be measured by cheap sensors with high accuracy on-line. The reality is more complicated: there are some parameters that can be reliably measured by relatively cheap sensors (temperature, pO_2_, pH, O_2_, methanol). At the same time, most parameters or exchange fluxes of metabolites cannot be measured online or at-line at all.

As part of automatic search in the combinatorial space of possible designs, the complexity of marker rule detection needs to be compared with a criterion to enable ranking of the top designs. To enable assignment of a numerical value to the inclusion of a sensor, a list of measurable parameters should be developed where each sensor obtains a score that depends on the costs of the measurement (including maintenance and other costs), the accuracy of the measurement and relevance of the measurement. For instance, a biomass measurement may be less relevant if it counts both live and dead cells compared with a sensor that counts just living cell. A *SensorScore* is introduced to account for the different features of measurable parameters. *SensorScore* must be minimized. It takes into account the cost of sensors (*Cost*, lower is better), accuracy of sensors (*Accuracy*, lower number means higher accuracy) and the relevance (how much the measurement can tell about the exchange flux) of the measurement (*Relevance*, higher value means better):

*SensorScore* = *W_cost_* × *Cost* + *W_accuracy_* × *Accuracy* − *W_reliability_* × *Relevance*

where *W_cost_*, *W_accuracy_* and *W_relevance_*—weight factors for sensor characteristics *Cost*, *Accuracy* and *Relevance*, correspondingly. A smaller *SensorScore* means it is better.

*Cost*, *Accuracy* and *Relevance* can be expressed in arbitrary units, which can be added up by weight factors. A smaller number of sensors installed results in a smaller sum of *SensorScores*. *SensorScore* is one component used to rank designs. Higher summary *SensorScore* of all parameters contained in the marker rule reduces design attractiveness.

### 4.2. Growth Coupling as a Good Starting Point

The reduction of flux variability of parameters, which have to be measured in the desired steady state to uniquely identify the state, is the key towards determination of marker rules. The growth coupling approach described on the example of *E. coli* described in Section 3.2. is a good way to illustrate its applicability in marker design. The variety of growth coupled design tools [22] facilitate the application of growth coupling.

It is also possible to design coupling between different fluxes without relating to growth. Biomass can be considered just as one of exchange metabolites contributing to marker rule composition. Biomass measurement may be replaced by other sensors during strain and marker rule design. Online biomass measurements could be avoided that way.

### 4.3. Ranking of Designs with Marker Rules

As mentioned at the beginning of Section 4, development of marker enabling strain design is a complex task with a huge combinatorial solution space of variables. Search of the best design, therefore, has to be automated and handed over to methods that are specialized in search of the best design in the solution space like evolutionary algorithms [31], minimal cut sets (MCS) [33], MILP methods [32,34,35] or some combination of previous methods like StrainDesign [36]. Handing design evaluation to software implies definition of optimization criterion or objective function.

We propose to use an objective function (*Obj*) that must be maximized and consists of sum of weighed parameters that characterize (1) productivity/yield, taking into account the number of necessary reaction deletions/insertions (*Prod*) and (2) the sum of *SensorScore*:

*Obj* = *W_Prod_* × *Prod* − *W_SensorScore_* × SUM(*SensorScore*),

where *W_Prod_*, *W_SensorScore_*—weights of different objective function components used to compensate different dimensions of components and assign different importance to them, SUM—operator of summation.

The relative influence of different components of the objective function can be brought into the right proportion by weight coefficients to observe the versatility of the generated designs. This is because a dominance of one objective function component may not be desired. The proposed objective function can be enriched with additional parameters with a dedicated weight coefficient to balance the influence of the new parameter the way that it obtains appropriate impact.

## 5. Decision Support in Case of Shift from the Desired Fermentation State by Marker Rules

Further development of automatically detectable steady state marker rules would be not just for identification of the current fermentation state, but also for recognition of deviation from the desired steady state at an early stage and suggest the most appropriate correction action that may include the termination of the fermentation process.

**Figure 4:**
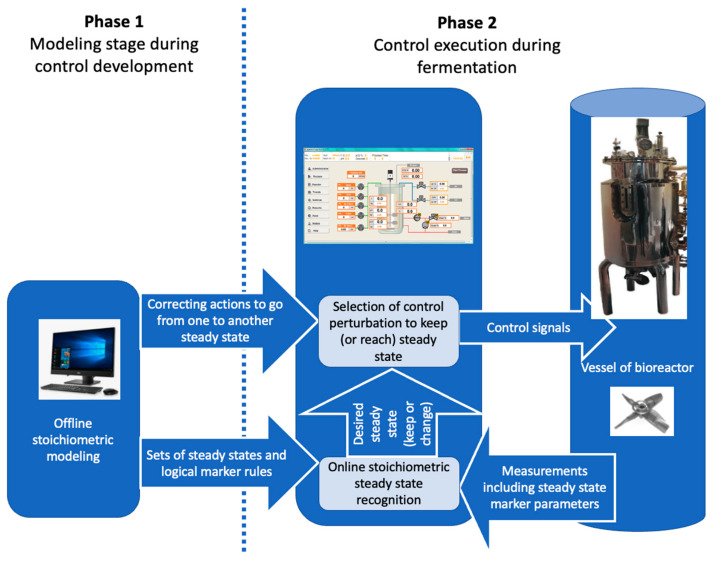
An implementation of a two-phase marker rule concept for detecting the desired steady state and correcting deviations.

In an industrial setting, the marker-based control system can be integrated into the bioreactor control system (Figure 4). In order to avoid continuous involvement of a modeler, we propose to perform modeling-related activities during Phase 1 (Figure 4) while designing a certain biotechnological production system. The outcome of Phase 1 would be marker rules and correcting action algorithms to counteract the deviation from desired steady state and return the process back to the desired one. The return to the desired steady state may be achieved by changing concentrations of metabolites in the fermentation broth or changing fermenter settings (aeration rate, gas content, mixing rate, pH). This way, there would be no need for a modelling specialist during production (Phase 2) as the rules derived from modelling (Phase 1) would be embedded in the control algorithm of the bioreactor control system and Phase 2 (actual control of bioprocess) takes place automatically. This means that marker rules are part of the strain design documentation.

Some of correction the rules might suggest finishing the fermentation if the particular deviation to an alternative steady state cannot be prevented and the economically best strategy is to end the fermentation process.

## 6. Discussion

Creating measurable and automatically recognizable markers of desired steady state is a new approach to metabolism engineering using genome scale stoichiometric metabolic engineering. The use of model-based correction strategies to avoid the shift from the desired steady state (Section 5) becomes valuable or even irreplaceable in the case of unstable processes. It is important that marker rules are derived from genome-scale mechanistic models of cellular metabolism in contrast to soft sensors, where black-box approaches dominate [2,3,37]. A mechanistic explanation of marker rules also enables better chances to interpret deviations from the rule, and may lead to improvements in the genome scale model if necessary.

Using this type of advanced bioreactor control system, metabolic modelling specialists do not need to be present on site since the fermentation can still benefit from the modeling results performed during strain design (Figure 4).

Different factors considered as variables in this study can influence the value of a design. The chassis organism is very important, because of different metabolic strategies available or absent in the strain. Pathways that should be implemented in one strain may be available in another one. The same is with deletions: some strains may not have pathways that should be deleted in another strain. A high acid resistance for large scale industrial applications is an important feature [38,39] that can be implemented in the design, selecting acid-resistant chassis strains for a measurable steady state marker rule in metabolic engineering. Other important features that may not be identified by metabolic engineering can be brought into the design by selecting a chassis strain with necessary features.

Another factor with game-changing potential is the development of measurement technologies and systems. A technological progress of on-line measurement of a specific metabolite can change the rank of designs, as well as the attractivity of particular chassis strains in general.

It is possible that there are processes where no feasible markers of metabolism can be found due to the peculiarities of the product or the metabolic network of the organism.

In the case of practical implementation, there are expected problems with measurement errors and some parameter “noise” during fermentation. On the other hand, at the stage where a number of fermentations have been performed and measurement time-series are available, artificial intelligence and machine learning may be applied to recognize early stages of steady state shift by the dynamic features of some measurements made to test the marker rules.
